# Active case finding among marginalised and vulnerable populations reduces catastrophic costs due to tuberculosis diagnosis

**DOI:** 10.1080/16549716.2018.1494897

**Published:** 2018-09-03

**Authors:** Hemant Deepak Shewade, Vivek Gupta, Srinath Satyanarayana, Atul Kharate, K.N. Sahai, Lakshmi Murali, Sanjeev Kamble, Madhav Deshpande, Naresh Kumar, Sunil Kumar, Prabhat Pandey, U.N. Bajpai, Jaya Prasad Tripathy, Soundappan Kathirvel, Sripriya Pandurangan, Subrat Mohanty, Vaibhav Haribhau Ghule, Karuna D. Sagili, Banuru Muralidhara Prasad, Sudhi Nath, Priyanka Singh, Kamlesh Singh, Ramesh Singh, Gurukartick Jayaraman, P. Rajeswaran, Binod Kumar Srivastava, Moumita Biswas, Gayadhar Mallick, Om Prakash Bera, A. James Jeyakumar Jaisingh, Ali Jafar Naqvi, Prafulla Verma, Mohammed Salauddin Ansari, Prafulla C. Mishra, G. Sumesh, Sanjeeb Barik, Vijesh Mathew, Manas Ranjan Singh Lohar, Chandrashekhar S. Gaurkhede, Ganesh Parate, Sharifa Yasin Bale, Ishwar Koli, Ashwin Kumar Bharadwaj, G. Venkatraman, K. Sathiyanarayanan, Jinesh Lal, Ashwini Kumar Sharma, Raghuram Rao, Ajay M.V. Kumar, Sarabjit Singh Chadha

**Affiliations:** a Department of Operational Research, International Union Against Tuberculosis and Lung Disease (The Union), South-East Asia Office, New Delhi, India; b Centre for Operational Research, International Union Against Tuberculosis and Lung Disease (The Union), Paris, France; c Dr. Rajendra Prasad Centre for Ophthalmic Sciences, All India Institute of Medical Sciences (AIIMS), New Delhi, India; d State TB Cell, Department of Health & Family Welfare, Government of Madhya Pradesh, Bhopal, India; e State TB Cell, Department of Health & Family Welfare, Government of Bihar, Patna, India; f State TB Cell, Department of Health & Family Welfare, Government of Tamil Nadu, Chennai, India; g State TB Cell, Health Department, Government of Maharashtra, Pune, India; h State TB Cell, Department of Health & Family Welfare, Government of Chattisgarh, Raipur, India; i State TB Cell, Department of Health & Family Welfare, Government of Punjab, Chandigarh, India; j State TB Cell, Department of Health & Family Welfare, Government of Kerala, Thiruvananthapuram, India; k Department of TB and Communicable Diseases, International Union Against Tuberculosis and Lung Disease (The Union), New Delhi, India; l Voluntary Health Association of India (VHAI), New Delhi, India; m Department of Community Medicine, Post Graduate Institute of Medical Education and Research (PGIMER), Chandigarh, India; n MAMTA Health Institute for Mother and Child, New Delhi, India; o Catholic Health Association of India (CHAI), Telangana, India; p Resource Group for Education & Advocacy for Community Health (REACH), Chennai, India; q Population Services International (PSI), New Delhi, India; r Catholic Bishops’ Conference of India-Coalition for AIDS and Related Diseases (CBCI-CARD), New Delhi, India; s Emmanuel Hospital Association (EHA), New Delhi, India; t Central TB Division, Revised National Tuberculosis Control Programme, Ministry of Health and Family Welfare, Government of India, New Delhi, India

**Keywords:** tuberculosis/prevention and control, systematic screening, vulnerable populations, health care costs, health equity

## Abstract

**Background**: There is limited evidence on whether active case finding (ACF) among marginalised and vulnerable populations mitigates the financial burden during tuberculosis (TB) diagnosis.

**Objectives**: To determine the effect of ACF among marginalised and vulnerable populations on prevalence and inequity of catastrophic costs due to TB diagnosis among TB-affected households when compared with passive case finding (PCF).

**Methods**: In 18 randomly sampled ACF districts in India, during March 2016 to February 2017, we enrolled all new sputum-smear-positive TB patients detected through ACF and an equal number of randomly selected patients detected through PCF. Direct (medical and non-medical) and indirect costs due to TB diagnosis were collected through patient interviews at their residence. We defined costs due to TB diagnosis as ‘catastrophic’ if the total costs (direct and indirect) due to TB diagnosis exceeded 20% of annual pre-TB household income. We used concentration curves and indices to assess the extent of inequity.

**Results**: When compared with patients detected through PCF (*n* = 231), ACF patients (*n* = 234) incurred lower median total costs (US$ 4.6 and 20.4, *p* < 0.001). The prevalence of catastrophic costs in ACF and PCF was 10.3 and 11.5% respectively. Adjusted analysis showed that patients detected through ACF had a 32% lower prevalence of catastrophic costs relative to PCF [adjusted prevalence ratio (95% CI): 0.68 (0.69, 0.97)]. The concentration indices (95% CI) for total costs in both ACF [−0.15 (−0.32, 0.11)] and PCF [−0.06 (−0.20, 0.08)] were not significantly different from the line of equality and each other. The concentration indices (95% CI) for catastrophic costs in both ACF [−0.60 (−0.81, –0.39)] and PCF [−0.58 (−0.78, –0.38)] were not significantly different from each other: however, both the curves had a significant distribution among the poorest quintiles.

**Conclusion**: ACF among marginalised and vulnerable populations reduced total costs and prevalence of catastrophic costs due to TB diagnosis, but could not address inequity.

## Background

Tuberculosis (TB) is the leading cause of death among infectious diseases. In 2016, an estimated 10.4 million people developed TB, and 1.7 million died from it [1]. Despite TB diagnosis and treatment services being free under the national TB programmes, patients incur significant direct medical, direct non-medical and indirect costs due to TB care [2]. Measuring costs especially during diagnosis of TB is important because it is the most uncertain period during illness, and most of the social protection measures do not cover costs incurred during diagnosis [3–7]. A systematic review reported that the total cost due to TB care was equivalent to 39% (range: 4–148%) of the annual household income (AHI). Half of the total cost was incurred before TB treatment [8].

High costs due to TB diagnosis could be because of the way in which TB care services are organised. Patients have to visit health services on their own for diagnosis [passive case finding (PCF)], and only after TB diagnosis does the programme takes up active responsibility to care for them [9]. The process to reach the health facilities could be time-consuming, cumbersome and costly [10–12]. As TB services are integrated with the general health system, the geographical, financial and social access barriers to TB care are similar to the barriers of accessing the general health system [4,13]. Patients get trapped in a vicious circle of repeated visits to the same health care provider (HCP) or visits to multiple HCPs including private and traditional HCPs [8,10,11,14,15].

The World Health Organization’s End TB strategy envisages that by 2035, no TB-affected household should incur catastrophic costs due to TB care. One of its four principles is to ensure the protection and promotion of human rights, ethics and equity. Systematic screening of those at high risk for TB is a key component of the End TB strategy. Active case finding (ACF) can reduce the costs due to TB diagnosis through early case detection [3,9].

India accounted for 27% of the estimated global TB burden in 2016, which included 0.4 million TB deaths [1,16]. In line with the strategic vision of India’s revised national TB control programme (RNTCP) (2012–2017) [17], project *Axshya* (meaning ‘free of TB’) was implemented by the South-East Asia office of the International Union Against Tuberculosis and Lung Disease (The Union) [18–20]. *Axshya SAMVAD* (Sensitisation and Advocacy in Marginalised and Vulnerable Areas of the District) is the ACF strategy under the project. *SAMVAD* in Sanskrit language means ‘conversation’. It resulted in the detection of a large number of persons with presumptive pulmonary TB and sputum-smear-positive TB [21]. However, whether it mitigated the financial impact of the disease on the patient’s households is unknown [9,22].

Globally, there is only one study that assessed the effect of ACF (screening household and neighbourhood contacts) on catastrophic costs due to TB (Cambodia in 2012–2013); however, no adjusted analysis was performed. The effect of ACF on inequity in distribution of catastrophic costs was also not assessed [23]. Although there have been studies on patient costs due to TB care in India [24], studies on catastrophic costs are limited.

Hence, this study was conducted to determine the effect of ACF in marginalised and vulnerable populations on catastrophic costs (prevalence, intensity and inequity) due to TB diagnosis among TB-affected households when compared with PCF alone.

## Methods

### Study design

This was an observational analytic study involving primary as well as secondary data collection.

### Study setting

#### India’s national TB programme

RNTCP infrastructure includes national-, state-, district- and sub-district-level administrative units. The district TB centres, sub-district level programme management units called as TB units (TUs – one for 250 000 to 500 000 population) and designated microscopy centres (DMCs – one for 50 000 to 100 000 population) for sputum microscopy work under the administrative control of the State TB officer. Patients with presumptive TB visit the DMCs for sputum examination and diagnosis. Laboratory registers maintained in each DMC contain details of each person who underwent sputum smear microscopy, and TB registers maintained at each TU indicate the number of TB patients treated under RNTCP [25]. TB diagnosis and treatment services under RNTCP are provided free of charge.

#### Axshya SAMVAD under project Axshya *during 2016–2017*

Funded by The Global Fund against AIDS, TB and Malaria, the goal of project *Axshya* was to enhance the reach and visibility of RNTCP services among marginalised and vulnerable population and mitigate the impact of TB [18–20]. In consultation with the State TB programme, *Axshya* districts (total of 285 districts in 18 states) and *Axshya* TUs were identified. Within an *Axshya* TU, activities (including *Axshya SAMVAD*) were preferentially targeted towards marginalised and vulnerable populations (see S1 Annex for the criteria used to define marginalised and vulnerable populations). Each *Axshya* district had a district coordinator (DC) who was supervised by the assistant project manager, state technical consultant and project management unit at The Union South East Asia office in New Delhi, India.

Technical and operational guidelines for *Axshya SAMVAD* (2016–17) are provided in S1 Annex. Community volunteers, trained by DC, conducted house-to-house visits to create awareness about TB, identified presumptive TB patients (people with any one of: >2 weeks of cough, fever, loss of appetite, loss of weight) and referred to the nearest DMC for sputum examination. Sputum collection and transport were carried out after a documented ‘failed referral’ [18].

### Study population and sampling

**Figure 1.**
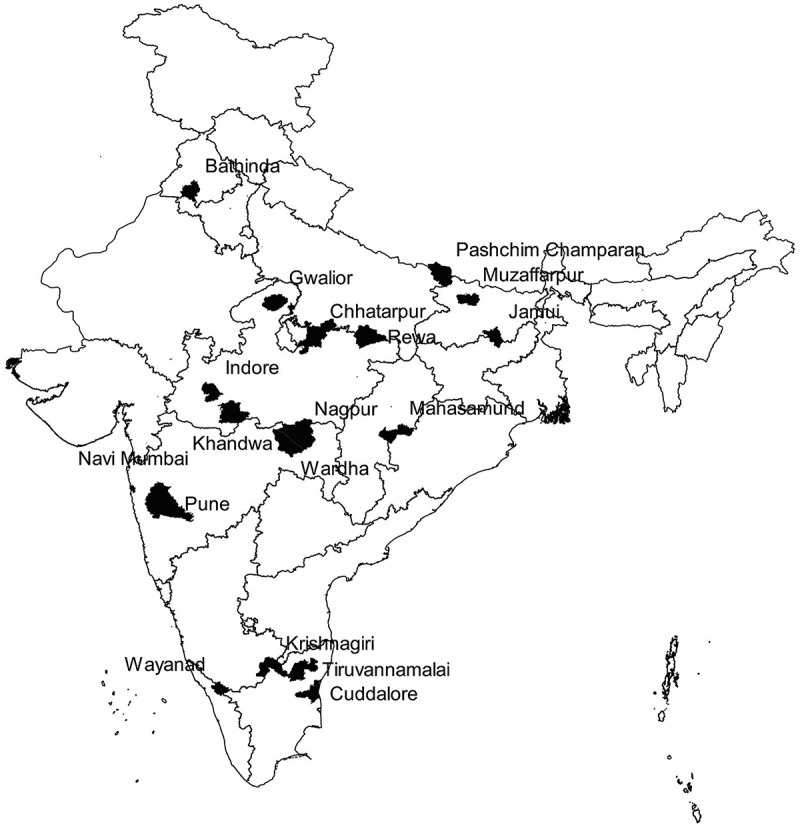
Map of India depicting the randomly sampled *Axshya* districts (*n* = 18) under *Axshya SAMVAD* study, India (2016–17) [26]. *SAMVAD: Sensitisation and Advocacy in Marginalised and Vulnerable Areas of the District. *Axshya SAMVAD*: an active case finding strategy under project *Axshya* implemented by The Union, South East Asia office, New Delhi, India, across 285 districts of India. *Reprinted with permission of the International Union Against Tuberculosis and Lung Disease. © The Union [26].

All sputum-smear-positive TB patients newly registered for treatment between March 2016 and February 2017 and belonging to marginalised and vulnerable population in *Axshya* districts constituted the study population. We randomly sampled (simple random sampling) 18 districts from the 285 Axshya districts of India considering the feasibility of data collection. The sampling frame for these districts excluded districts from north-eastern India (due to the difficulty in the logistics of collecting data in the hilly terrain). These 18 districts belonged to seven states (Figure 1).

At the beginning of every month (from April 2016 to March 2017) in every study district, the DC prepared a line list of new sputum-smear-positive TB patients registered in the previous month. This was compiled at district level, and each patient was provided a unique identifier (state code–district code–TU code–year–registration number). The patients were classified into three groups and updated in the open-access tool-based model: exposed; unexposed and eligible; and unexposed but ineligible [26].

The operational definition for each group is summarised in Table 1. To summarise, the ‘exposed’ group included patients identified through ACF (*Axshya SAMVAD*), and the ‘unexposed’ group included patients that were identified by PCF (non-*Axshya SAMVAD*). ‘Unexposed and ineligible’ group contained patients with mixed or contaminated exposure: in other words, these patients were identified through PCF and Axshya SAMVAD activity was conducted in their village before the date of diagnosis [26].

**Table 1.** Operational definition of study participants and sampling methodology in *Axshya SAMVAD* study, India (2016–17) [26]^a^.

Terminology	Definition
Study participant	New sputum-smear-positive TB patients registered for treatment and belonging to a marginalised and vulnerable population in the district
Study participant – exposed	New sputum-smear-positive TB patients diagnosed through *Axshya SAMVAD*, i.e. participants’ residence belongs to a village/urban ward where *Axshya SAMVAD* was conducted before the date of diagnosis, and there is clear documentation in the project records that the patient was identified by *Axshya SAMVAD*
Study participant – unexposed and eligible	New sputum-smear-positive TB patients (detected through passive case finding) and belong to a village/urban ward where *Axshya SAMVAD was not conducted* (ever) before the date of diagnosis
Study participant – unexposed and ineligible	New sputum-smear-positive TB patients (detected through passive case finding) but belonged to a village where *Axshya SAMVAD was conducted* (ever) before the date of diagnosis. In such patients, it was challenging to rule out exposure to *Axshya SAMVAD*, and hence they were excluded from the study
Sampling	All the ‘exposed’ were enrolled into the study, an equal number from the list ‘unexposed and eligible’ were randomly enrolled as ‘unexposed’ (1:1 ratio, exposed: unexposed), and all the ‘unexposed but ineligible’ were excluded from the study

TB: tuberculosis; *SAMVAD*: sensitisation and advocacy in marginalised and vulnerable areas of the district; *Axshya SAMVAD*: an active case finding strategy under project *Axshya* implemented by The Union, South East Asia office, New Delhi, India, across 285 districts of India.

^a^Reprinted with modification with permission of the International Union Against Tuberculosis and Lung Disease. Copyright © The Union [26].

All the ‘exposed’ patients were enrolled into the study. An equal number from the list ‘unexposed and eligible’ were randomly (simple random sampling) enrolled as ‘unexposed’ (1:1 ratio, exposed: unexposed), and all the ‘unexposed but ineligible’ were excluded from the study [26].

### Data collection

Data were collected between April 2016 and June 2017. Details of data-collection procedure, monitoring of data collection and quality control have been described elsewhere [26].

The questionnaire used for data collection was divided into two parts. Part I contained variables that were extracted by reviewing the TB treatment register, treatment card and project *Axshya* records (S2 Annex). Part II was an interviewer-administered, structured, closed-ended questionnaire administered during a subsequent residential visit (S3 Annex). The pre-TB AHI and number of family members were used to calculate the monthly income per capita (MIPC) at the time of data collection. Information on costs incurred were collected for every consultation between eligibility for sputum examination [fifteenth day of continuous cough or fever or the day of the first episode of haemoptysis (whichever was earlier)] and date of diagnosis. This included direct medical costs (consultation fee, investigations and medicines), direct non-medical costs (diagnosis-related transport) and indirect costs (patient’s income loss for the time spent on consultation).

We did not include information on costs after diagnosis (before treatment and during treatment) because all patients irrespective of whether they were detected through ACF or PCF received the same treatment under RNTCP.

### Analysis and statistics

#### Data management

Data collected were double entered, validated and analysed during July to December 2017 using EpiData software (version 3.1 for entry and version 2.2.2.183 for analysis; EpiData Association, Odense Denmark). An equity assessment and adjusted analysis were performed using STATA (version 12.1, copyright 1985–2011 StataCorp LP USA).

For classification for below the poverty line, we used the Indian MIPC cut-off of 972 INR (~US$ 15.3) and 1407 INR (~US$ 22.1) for rural and urban areas, respectively [27]. The results are presented in US$ using the average conversion rate of US$1 = 63.6 INR (January 2018).

We summarise below the details of data analysis. However, for further details on analysis, the readers may wish to refer to previous references [28–30].

#### Cost description, prevalence and intensity of catastrophic costs

Direct medical, direct non-medical, indirect and total costs (overall, public private) were described (in US$) using the median and interquartile range (IQR). Based on the MIPC, assuming 22 working days per month and eight working hours per day, we calculated the value in terms of money for each hour spent for consultation (indirect costs).

The total costs for TB diagnosis were calculated and defined as catastrophic if they exceeded 20% of pre-TB AHI [2]. Data on pre-TB AHI and number of family members were rarely recorded (in Part II of questionnaire). Hence, during the analysis, we used the average household size of 4.8 in the seven states (to which these 18 districts belonged) to derive the pre-TB AHI [31]. The intensity of catastrophic costs was measured as median-positive overshoot beyond the 20% threshold [30]. Positive overshoot was calculated among those with catastrophic costs by subtracting 20% from the total costs expressed as a proportion of annual pre-TB household income [30].

The operational definitions of various costs and indicators used are summarised in Box 1.

#### *Effect of* Axshya SAMVAD *on catastrophic costs due to TB diagnosis*

We did a confounder-adjusted analysis (causal modelling) for the association between *Axshya SAMVAD* (yes – exposure) and catastrophic costs due to TB diagnosis (yes – outcome) using log binomial regression after adjusting for clustering at district level. A complete case analysis was performed. Variables were considered as potential confounders if they were associated with exposure (*p* < 0.05 or programmatically or clinically significant difference) and outcome (*p* < 0.20) (for details, see Table S1). Age and gender were considered as potential confounders irrespective of the *p* values. A potential confounder was retained in the model as a confounder if removing it resulted in a change in the beta coefficient for the exposure (*Axshya SAMVAD*) by more than 15% [32]. We did not adjust for the number of HCPs visited and the type of first HCP visited. We hypothesised that these were in the causal pathway between exposure and outcome and did not qualify as confounders. Association was summarised (inferred) using unadjusted and adjusted prevalence ratios (95% CI).

#### Assessment of equity

Income quintiles were generated by ranking the households based on MIPC. The distribution of total costs due to TB diagnosis were summarised across income quintiles as follows: (1) absolute total costs, (2) total costs as a proportion of pre-TB AHI and (3) proportion of households experiencing catastrophic costs.

Concentration curves and concentration indices (along with 95% CI) were used to assess the extent of inequity in the distribution of all the above three indicators (in y axis) against cumulatively ranked households (poorest to richest – x axis). The values of concentration indices range from + 1 to –1; with positive values (concentration curve below the line of equality) suggesting disproportionate concentration among the rich, and negative values (concentration curve above the line of equality) suggesting disproportionate concentration among the poor [29,30]. For the indicator ‘total costs’, we assumed equity if the concentration curve and index revealed a significant distribution across the richest quintiles (positive concentration index, 95% CI not including zero). For the indicators, ‘total costs as a proportion of pre-TB AHI’ and ‘catastrophic costs’, we assumed equity if the concentration curve and index revealed an equal distribution across the quintiles (concentration curve not significantly different from the line of equality). A comparison of concentration curves across *Axshya SAMVAD* and non-*Axshya SAMVAD* groups was performed using dominance tests [30].

## Results

Of 661 enrolled, 88 were excluded later, as it was found that they did not fit into the study participant definition. Of 573 eligible, patient interviews were not conducted for 108 due to patient non-availability during their visit to the residence. When compared with those interviewed (*n* = 465), those not interviewed (*n* = 108) were less likely to be patients registered through *Axshya SAMVAD*, from rural areas and with a sputum grading of 3+ at diagnosis (Table S2).

The 465 patients with completed interviews were included in the final analysis: 234 in the *Axshya SAMVAD* group and 231 in the non-*Axshya SAMVAD* group. The time taken between enrolment and interview is summarised in Table S3. Of 465, only one (<1%) was living with HIV, while the HIV status was unknown for 177 (38%) patients.

### Baseline characteristics

The socio-demographic, clinical and health system level characteristics of the *Axshya SAMVAD* and non-*Axshya SAMVAD* groups are summarised in Table 2. Compared with the non-*Axshya SAMVAD* group, in the *Axshya SAMVAD* group the median MIPC was lower (US$ 15.7 versus 13.1, *p* = 0.014), and the proportion of households below the poverty line was higher (45.4% versus 51.9%, *p* = 0.19).

**Table 2.** Baseline characteristics of patients with new sputum-smear-positive TB enrolled in *Axshya SAMVAD* study across 18 randomly sampled districts in India, 2016–17 (*n* = 465).

		*Axshya SAMVAD* group	Non-*Axshya SAMVAD* group	
	Total [*N* = 465]	[*N* = 234]	[*N* = 231]	
Variable	*n* (%)	*n* (%)	*n* (%)	*p* value^a^
Socio-demographic characteristics				
Age (years)				
15–44	251 (54)	111 (47)	140 (61)	0.009
45–64	163 (35)	91 (39)	72 (31)	
≥65	50 (11)	32 (14)	18 (8)	
Missing	1 (<1)	0 (0)	1 (<1)	
Mean (SD)	42 (17)	44 (17)	40 (17)	0.003
Gender				
Male	307 (66)	153 (65)	154 (67)	0.721
Female	157 (34)	81 (35)	76 (33)	
Missing	1 (<1)	0 (0)	1 (<1)	–
Residence				
Urban	58 (12)	17 (7)	41 (18)	<0.001
Rural	402 (87)	214 (92)	188 (81)	
Missing	5 (1)	3 (1)	2 (1)	
Education				
No formal education	217 (47)	133 (57)	84 (36)	<0.001
Less than primary	67 (14)	30 (13)	37 (16)	
Up to secondary	149 (32)	57 (24)	92 (40)	
Higher secondary and above	30 (7)	13 (6)	17 (7)	
Missing	2 (<1)	1 (<1)	1 (<1)	
Occupation				
Unemployed	59 (13)	31 (13)	28 (12)	0.283
Studying	24 (5)	8 (3)	16 (7)	
Homemaker	82 (18)	45 (19)	37 (16)	
Daily wage labour	178 (38)	95 (41)	83 (36)	
Employed – not daily wage	113 (24)	52 (22)	61 (26)	
Missing	9 (2)	3 (1)	6 (3)	
Monthly income per capita ($US)^b^				
Median (IQR)	15.7 (7.4, 31.4)	13.1 (6.4, 23.6)	15.7 (7.9, 31.4)	0.014
Clinical characteristics				
TB in household in the past				
Yes	116 (25)	54 (23)	62 (27)	0.321
No	347 (75)	180 (77)	167 (72)	
Missing	2 (<1)	0 (0)	2 (1)	
TB death in the household				
Yes	51 (11)	27 (11)	24 (10)	0.704
No	413 (89)	207 (89)	206 (89)	
Missing	1 (<1)	0 (0)	1 (<1)	
History of fever^c^				
Yes	350 (75)	170 (73)	180 (78)	0.231
No	105 (22)	58 (25)	47 (20)	
Missing	10 (3)	6 (2)	4 (2)	
History of weight loss^c^				
Yes	340 (73)	159 (68)	181 (78)	0.032
No	113 (24)	66 (28)	47 (20)	
Missing	12 (3)	9 (4)	3 (2)	
History of haemoptysis^c^				
Yes	119 (26)	60 (25)	59 (26)	0.937
No	336 (72)	168 (72)	168 (73)	
Missing	10 (2)	6 (3)	4 (1)	
Current smoker^d^				
Yes	113 (24)	65 (28)	48 (21)	0.122
No	343 (74)	164 (70)	179 (77)	
Missing	9 (2)	5 (2)	4(2)	
Current alcohol intake^d^				
Yes	130 (28)	61 (26)	69 (30)	0.419
No	327 (70)	168 (72)	159 (69)	
Missing	8 (2)	5 (2)	3 (1)	
Sputum grading				
3+	83 (18)	34 (15)	49 (21)	0.068
Scanty/1+/2+	365 (79)	190 (81)	175 (76)	
Positive not quantified	17 (4)	10 (4)	7 (3)	
Weight (kg)				
<30	8 (2)	6 (2)	3 (1)	0.540
30–44.9	200 (43)	102 (44)	98 (42)	
≥ 45	96 (21)	44 (19)	52 (23)	
Missing	161 (35)	83 (35)	78 (34)	
Mean (SD)	41 (7)	41 (6)	41 (7)	0.781
HIV status^e^				
Positive	1 (<1)	0 (0)	1 (<1)	–
Negative	287 (59)	143 (61)	144 (62)	
Missing	177 (38)	91 (39)	86 (37)	
DM status				
DM	9 (2)	4 (2)	5 (2)	0.784
Not DM	171 (37)	84 (36)	87 (38)	
Missing	285 (61)	146 (62)	139 (60)	
Health-system characteristics				
Distance of residence from DMC in kilometre				
≤ 5	118 (25)	50 (21)	68 (29)	0.063
6–10	144 (31)	80 (34)	64 (28)	
11–15	107 (23)	49 (21)	58 (25)	
> 15	96 (21)	55 (24)	41 (18)	
Median (IQR)	10 (5,15)	10 (6, 15)	10 (5, 14)	0.090

Column percentage.

TB: tuberculosis; *SAMVAD*: sensitisation and advocacy in marginalised and vulnerable areas of the district; SD: standard deviation; HIV: human immunodeficiency virus; DM: diabetes mellitus; DMC: designated microscopy centre; IQR: interquartile range. *Axshya SAMVAD*: an active case finding strategy under project *Axshya* implemented by The Union, South East Asia office, New Delhi, India, across 285 districts of India.

^a^ *p* value calculated after excluding missing values, chi square test/independent *t* test/Mann–Whitney *U* test.

^b^Average Indian rupee to USD conversion rate in January 2018 (US$1 = 63.6 Indian rupees), Indian rupee value used for calculating *p* value.

^c^History of fever/significant weight loss/haemoptysis between eligibility for sputum examination and diagnosis.

^d^Consumption of alcohol/smoke form of tobacco anytime in the month before date of diagnosis.

^e^Number with HIV very low (*n* = 1); hence, *p* value not calculated.

### Costs due to TB diagnosis

The median direct medical (US$ 3.3 versus 15.7, *p* < 0.001), direct non-medical (0.3 versus 1.9. *p* < 0.001), indirect (US$ 0.1 versus 0.6, *p* < 0.001) and total costs due to TB diagnosis (US$ 4.6 and 20.4, *p* < 0.001) were lower in the *Axshya SAMVAD* group than in the non-*Axshya SAMVAD* group; however, the difference was not statistically significant for total costs incurred when visiting the private sector (Table 3). The proportion of ‘zero’ total costs (13.4% and 32.5%, *p* < 0.001) and ‘zero’ costs within each subgroup was also significantly lower in the *Axshya SAMVAD* group (Table 3).

**Table 3.** Costs and time due to TB diagnosis, stratified by Axshya *SAMVAD* and non-Axshya *SAMVAD* groups, among patients with new sputum-smear-positive TB enrolled in Axshya *SAMVAD* study in India, 2016–2017.

	Overall^b^ (*N* = 465)	Axshya *SAMVAD* (*N* = 234)	Non-Axshya *SAMVAD* (*N* = 231)	
Costs ($US)^a^/time due to TB diagnosis	Median (IQR)	Median (IQR)	Median (IQR)	*p* value^c^
Direct medical costs	8.3 (0.0, 44.1)	3.3 (0.0, 31.5)	15.7 (0.8, 58.0)	<0.001
Consultation fee	0.0 (0.0,5.2)	0.0 (0.0,3.1)	1.5 (0.0,6.3)	<0.001
Medicines	2.4 (0.0,28.3)	0.0 (0.0,18.9)	5.9 (0.0,39.6)	0.005
Investigations	0.0 (0.0,4.7)	0.0 (0.0,1.3)	0.0 (0.0,7.9)	<0.001
Direct non-medical costs (travel)	1.3 (0.0,4.7)	0.3 (0.0,3.1)	1.9 (0.0, 7.0)	<0.001
Direct costs (all)	10.9 (0.2, 50.4)	4.2 (0, 39.5)	19.1 (2.1, 67.3)	<0.001
Indirect costs (wages/income lost)	0.3 (0.0,1.3)	0.1 (0.0,0.8)	0.6 (0.2,1.7)	<0.001
Total costs	12.5 (0.4, 52.6)	4.6 (0, 40.1)	20.4(3.8,68.8)	<0.001
Total costs – public	0.8 (0.0, 2.7)	0.4 (0.0,2.1)	1.1 (0.0, 3.1)	0.014
Total costs – private	20.0 (4.5,67.6)	15.9 (2.1, 58.2)	24.2 (6.2, 73.1)	0.090
Time spent in hours for consultation	5.0 (0.0,16.0)	2.0 (0.0,10.0)	8.0 (2.0,18.0)	<0.001
Zero time/costs due to TB diagnosis	%	%	%	*p* value^d^
Direct medical costs	32.4	43.0	21.4	<0.001
Consultation fee	50.0	59.6	40.2	<0.001
Medicines	44.9	50.9	38.8	0.010
Investigations	62.8	72.2	53.1	<0.001
Direct non-medical costs (travel)	34.8	44.3	25.0	<0.001
Direct costs (all)	24.0	33.0	14.7	<0.001
Indirect costs (wages/income lost)	29.6	39.7	19.5	<0.001
Total costs	23.0	32.5	13.4	<0.001
Time spent in hours for consultation	29.4	40.0	19.0	<0.001

Column percentage.

IQR: Interquartile range; TB: tuberculosis; *SAMVAD*: sensitisation and advocacy in marginalised and vulnerable areas of the district. *Axshya SAMVAD*: an active case finding strategy under project *Axshya* implemented by The Union, South East Asia office, New Delhi, India, across 285 districts of India. Non-*Axshya SAMVAD*: patients detected through passive case findings.

^a^Average Indian rupee to USD conversion rate in January 2018 (US$1 = 63.6 Indian rupees), Indian rupee value used for calculating the *p* value.

^b^Total costs information was available from all 465. However, details of time spent in consultation and costs for consultation, medicines, investigations and travel were not available for 11 patients. ^c^Mann–Whitney test.

^d^Chi square test.

When we compared each cost component as a proportion of total costs, the *Axshya SAMVAD* group incurred lower costs for investigations (8.6% versus 13.1%), higher costs for travel (15.7% versus 9.1%) and lower indirect costs (3.5% versus 6.2%) than the non-*Axshya SAMVAD* (Table 4).

**Table 4.** Contribution of each component of costs due to TB diagnosis as a proportion of total costs, stratified by *Axshya SAMVAD* and non-*Axshya SAMVAD* groups, among patients with new sputum-smear-positive TB enrolled in Axshya *SAMVAD* study in India, 2016–2017 (*N* = 465).

	Overall (N = 465)	Axshya *SAMVAD* (N = 234)	Non-Axshya *SAMVAD* (N = 231)
Costs due to TB diagnosis as a proportion of total costs	%	%	%
Total costs	100	100	100
Direct medical costs	83.3	80.8	84.7
Consultation fee	12.6	13.0	12.3
Medicines	59.3	59.2	59.3
Investigations	11.5	8.6	13.1
Direct non-medical costs (Travel)	11.4	15.7	9.1
Direct costs	94.7	96.5	93.8
Indirect costs (Wages/income lost)	5.3	3.5	6.2

Column percentage.

*SAMVAD*: sensitisation and advocacy in marginalised and vulnerable areas of the district. *Axshya SAMVAD*: an active case finding strategy under project *Axshya* implemented by The Union, South East Asia office, New Delhi, India, across 285 districts of India. Non-*Axshya SAMVAD*: patients detected through passive case findings.

Total costs information was available from all 465. However, details of time spent in consultation and costs for consultation, medicines, investigations and travel were not available for 11 patients.

### Prevalence and intensity of catastrophic costs due to TB diagnosis

Due to missing data for either total costs or MIPC in 14 patients, a total 451 patients were included in further analysis. The overall prevalence of catastrophic costs due to TB diagnosis [% (95% CI)] in *Axshya SAMVAD* and non-*Axshya SAMVAD* group was 10.3 (6.9, 14.9) and 11.5 (7.9, 16.3) respectively (Table 5), and the difference was not statistically significant (*p* = 0.064). The median (IQR) intensity of catastrophic costs (expressed as percentage overshoot beyond catastrophic threshold) in *Axshya SAMVAD* and non-*Axshya SAMVAD* group was 22 (5, 111) and 37 (9, 87) respectively (*p* = 0.703).

**Table 5.** Confounder-adjusted association between *Axshya SAMVAD* exposure and catastrophic costs due to TB diagnosis (outcome) using log binomial regression after accounting for clustering in districts, *Axshya SAMVAD* study, India, 2016–2017(*N* = 451)^a^.

	*Axshya SAMVAD*	Non-*Axshya SAMVAD*		
	% (outcome/total)	% (outcome/total)	PR (95% CI)	aPR (95% CI)^b^
Assuming average household size of 4.8	10.3 (23/224)	11.5 (26/227)	0.89 (0.56, 1.42)	0.68 (0.69, 0.97)^c^
Sensitivity analysis				
Assuming average household size of 3.9	12.9 (29/224)	14.5 (33/227)	0.89 (0.56, 1.41)	0.72 (0.53, 0.97)^c^
Assuming average household size of 5.5	8.5 (19/224)	10.1 (23/227)	0.84 (0.47, 1.49)	0.69 (0.50, 0.94)^c^

TB: tuberculosis; *SAMVAD*: sensitisation and advocacy in marginalised and vulnerable areas of the district; *Axshya SAMVAD*: an active case finding strategy under project *Axshya* implemented by The Union, South East Asia office, New Delhi, India, across 285 districts of India; Non-*Axshya SAMVAD*: patients detected through passive case findings; PR: prevalence ratio; aPR: adjusted prevalence ratio.

^a^Costs due to TB diagnosis were more than 20% of pre-TB annual household income.

^b^Complete case analysis was performed; model building (log binomial) by backward stepwise method. Age, sex, monthly income per capita, education, history of weight loss and distance of residence from microscopy centre were the confounders adjusted for.

^c^Statistically significant.

### Effect of Axshya SAMVAD on catastrophic costs

After adjusting for confounders (age, sex, patient education, MIPC, history of weight loss, distance of residence from nearest DMC), patients in the *Axshya SAMVAD* group had a 32% lower prevalence of catastrophic costs [aPR (95% CI): 0.68 (0.69, 0.97)]. Sputum smear grade, co-morbidities such as HIV and diabetes mellitus did not meet the criteria of potential confounders.

We also did a sensitivity analysis. We repeated the log binomial model, assuming a lower (3.9) and upper range (5.5) of household size in the seven states (to which these 18 districts belonged) [31]. *Axshya SAMVAD* remained significantly associated with lower catastrophic costs in both these models (Table 5).

### Equity assessment

**Figure 2.**
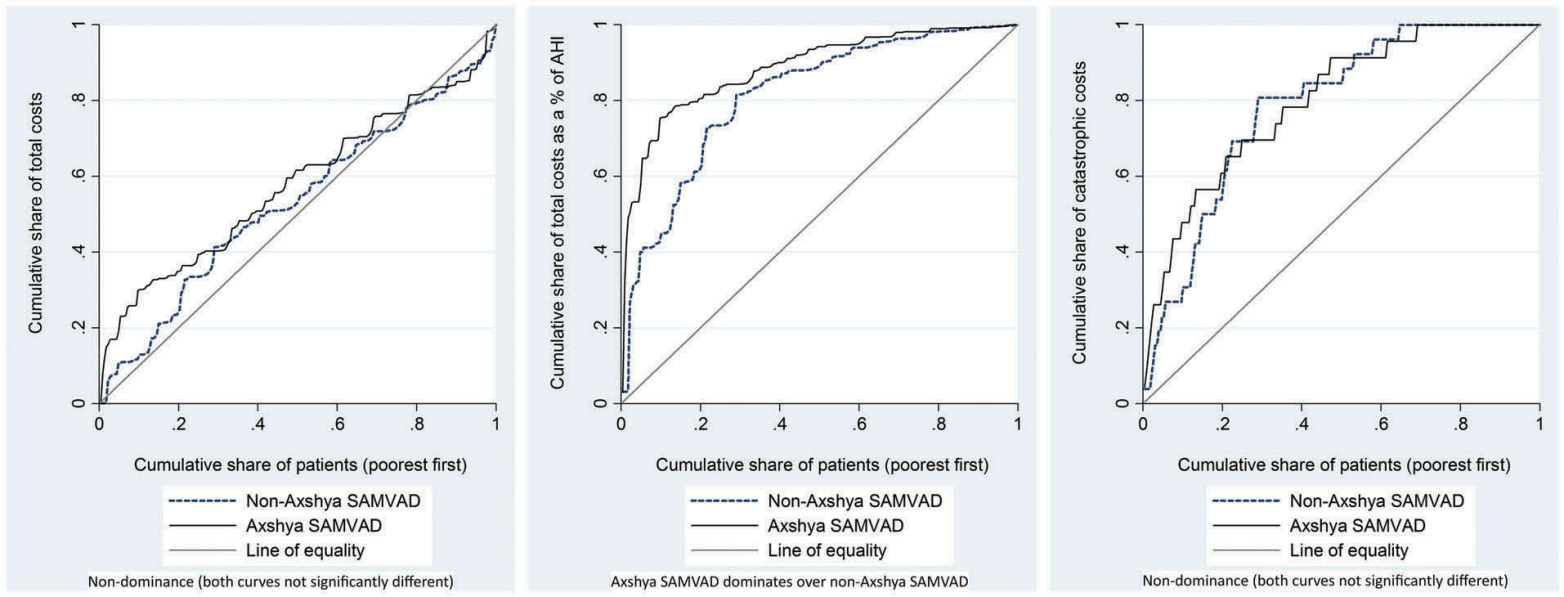
Concentration curves for total costs, total costs as a proportion of pre-TB annual household income and catastrophic costs due to TB diagnosis, stratified by Axshya *SAMVAD* and non-*Axshya SAMVAD* groups, among TB (new sputum-smear-positive) affected households in India, 2016–2017*. *SAMVAD*: sensitisation and advocacy in marginalised and vulnerable areas of the district; *Axshya SAMVAD*: an active case finding strategy under project *Axshya* implemented by The Union, South East Asia office, New Delhi, India, across 285 districts of India; TB: tuberculosis; AHI: annual household income. *For the indicator ‘total costs’, we assumed equity if the concentration curve/index revealed significant distribution across the richest quintiles (positive concentration index, 95% CI not including zero). For the indicators, ‘total costs as a proportion of pre-TB annual household income’ and ‘catastrophic costs’, we assumed equity if the concentration curve/index revealed equal distribution across the quintiles (concentration curve not significantly different from the line of equality). For details on the dominance test, readers are requested to refer to O’Donnell et al. [30].

Table 6 summarises the distribution of total costs, total costs as a percentage of pre-TB AHI and catastrophic costs due to TB diagnosis across income quintiles in *Axshya SAMVAD* and non-*Axshya SAMVAD* groups. In both the groups, the distribution of total costs did not vary significantly across income quintiles. The distribution of total costs as a percentage of pre-TB AHI and catastrophic costs was significantly higher in the poorest quintiles. This was confirmed by the corresponding concentration curves and indices (Figure 2 and Table 7).

**Table 6.** Distribution of total costs, total costs as a proportion of pre-TB annual household income and catastrophic costs due to TB diagnosis across income quintiles, stratified by *Axshya SAMVAD* and non-*Axshya SAMVAD* groups, among TB (new sputum-smear-positive) affected households in India, 2016–2017 (*N* = 451).

Characteristic	*Axshya SAMVAD* (*N* = 234)	Non-*Axshya SAMVAD* (*N* = 231)
Total costs	Median (IQR)	Median (IQR)
1st MIPC quintile	10.31 (0.13, 57.50)	16.89 (2.23, 61.94)
2nd MIPC quintile	5.28 (0, 37.81)	38.13 (7.72, 71.89)
3rd MIPC quintile	4.92 (0, 59.20)	18.00 (1.89, 69.40)
4th MIPC quintile	0.55 (0, 13.32)	17.99 (2.22, 59.37)
5th MIPC quintile	4.21 (0.27, 39.81)	22.17 (7.00, 77.22)
Overall	4.64 (0, 40.13)	20.36 (3.79, 68.76)
*p* value^a^	0.128	0.528
Total costs as a percentage of pre-TB annual household income	Median (IQR)	Median (IQR)
1st MIPC quintile	6.0 (0.0, 28.0)	8.0 (1.0, 36.5)
2nd MIPC quintile	1.0 (0.0, 9.0)	7.0 (1.0, 14.0)
3rd MIPC quintile	1.0 (0.0, 8.0)	2.0 (0.0, 6.8)
4th MIPC quintile	0.0 (0.0, 1.0)	1.0 (0.0, 3.3)
5th MIPC quintile	0.0 (0.0, 1.0)	1.0 (0.0, 2.0)
Overall	0.0 (0.0, 5.8)	2.0 (0.0, 8.0)
*p* value^a^	<0.001	<0.001
Catastrophic costs^b^	%	%
1st MIPC quintile	31.1	31.1
2nd MIPC quintile	8.9	15.6
3rd MIPC quintile	8.3	9.1
4th MIPC quintile	2.2	2.2
5th MIPC quintile	0	0
Overall	10.3	11.5
*p* value^a^	<0.001	<0.001

TB: tuberculosis; *SAMVAD*: sensitisation and advocacy in marginalised and vulnerable areas of the district; *Axshya SAMVAD*: an active case finding strategy under project *Axshya* implemented by The Union, South East Asia office, New Delhi, India, across 285 districts of India; non-*Axshya SAMVAD*: patients detected through passive case findings; MIPC: monthly income per capita.

^a^ *p* value to assess whether the distribution of the indicator was significantly different across the income quintiles (Kruskal–Wallis test for total costs and total costs as a percentage of pre-TB annual household income; chi square test for catastrophic costs).

^b^Total costs due to TB diagnosis more than 20% of pre-TB annual household income.

**Table 7.** Concentration indices for total costs, total costs as a proportion of pre-TB annual household income and catastrophic costs due to TB diagnosis, stratified by *Axshya SAMVAD* and non-*Axshya SAMVAD* groups, among TB (new sputum-smear-positive) affected households in India, 2016–2017 (*N* = 451).

	*Axshya SAMVAD*		Non-*Axshya SAMVAD*		
Characteristics	Concentration index (95% CI)	*p* value^a^	Concentration index (95% CI)	*p* value^a^	Dominance test [30]^b^
Total costs	−0.15 (−0.32. 0.11)	0.068	−0.06 (−0.20, 0.08)	0.401	Non-dominance (both curves not significantly different)
Total costs as a proportion of pre-TB annual household income	−0.77 (−1.14, −0.40)	<0.001	−0.63 (−092, −0.34)	<0.001	*Axshya SAMVAD* dominates over non-*Axshya SAMVAD*
Catastrophic costs^c^	−0.60 (−0.81, −0.39)	<0.001	−0.58 (−0.78, −0.38)	<0.001	Non-dominance (both curves not significantly different)

TB: tuberculosis; *SAMVAD*: sensitisation and advocacy in marginalised and vulnerable areas of the district; *Axshya SAMVAD*: an active case finding strategy under project *Axshya* implemented by The Union, South East Asia office, New Delhi, India, across 285 districts of India; non-*Axshya SAMVAD*: patients detected through passive case findings.

^a^ *p* value for the concentration index: indicates whether the concentration curve is significantly different from the line of equality.

^b^For details on the dominance test, readers are requested to refer to O’Donnell et al. [30].

^c^Total costs due to TB diagnosis more than 20% of pre-TB annual household income.

When compared with the non-*Axshya SAMVAD* group, the concentration index was more negative among the *Axshya SAMVAD* group for all three indicators (Table 7). For the indicator ‘total costs as a proportion of pre-TB AHI’, the *Axshya SAMVAD* concentration curve dominated over the non-*Axshya SAMVAD* curve. However, the concentration curves for catastrophic costs were not significantly different (Figure 2 and Table 7).

## Discussion

ACF among marginalised and vulnerable populations resulted in lower total costs and lower prevalence of catastrophic costs due to TB diagnosis. However, ACF did not address the issue of intensity and inequity in the distribution of catastrophic costs.

### Strengths

First, in addition to comparing the costs and their catastrophic impact, we also assessed inequity. The comparison group included patients from the same month and from the marginalised/vulnerable populations of the same area as the *Axshya SAMVAD* patients. Second, similar to Mauch et al. and Morishita et al. [11,23], we included a nationally representative sample, the reference population for which was limited to the *Axshya* districts (*n* = 285). The findings are also representative of their respective reference populations with potential contaminated exposures excluded. Third, we ensured quality control through audio recording of all the interviews followed by a random check by supervisors to reduce interviewer bias. We used an innovative resource-efficient model, which helped in near real-time data sharing and monitoring [26]. Finally, double data entry and validation minimised data-entry errors.

### Limitations

There were several major limitations. First, we did not enrol patients with initial loss to follow-up, and of those enrolled, we could not conduct interviews for 108 (19%). We compared patients and found that patients residing in rural areas and those with a high sputum grade were more likely to be excluded in our study (Table S2). It is likely that patients in rural areas and those with a high sputum grade may have incurred relatively more costs while undergoing diagnosis, and so our study results may be an underestimate of the key outcomes. Second, patients may have had challenges in recalling information on costs incurred. This recall limitation was non-differential, as it was similar in both the groups (similar delay from enrolment to interview; see Table S3).

Third, we derived the pre-TB AHI from MIPC by assuming an average household size of 4.8. However, while analysing the effect of *Axshya SAMVAD* on catastrophic costs, we also did a sensitivity analysis for varying household sizes. The findings were consistent and, thus, robust.

Finally, we were expecting most of the consultations for TB diagnosis to be for outpatients, and existing social insurance mechanisms in India do not cover for these. Similarly, food and stay costs are not expected to be significant for outpatient consultations. While we did not assess these parameters, we do not expect these to be differential among both the groups.

### Interpretation of key findings

Limitations notwithstanding, our study had many key findings. Costs (total and each component) were lower in the ACF group. ACF prevented one-third of the catastrophic costs due to TB diagnosis in all patients detected through ACF. These were possibly mediated through a lower number of visits to HCPs (median one versus two) and a higher proportion with zero visits (22% versus 0%) and first visit to an HCP in a public facility (42% versus 26%) in the *Axshya SAMVAD* group (data not shown). The costs due to TB diagnosis, total and each component, across ACF and PCF groups, were more or less similar to the findings of Morishita et al. (Table S4). Though the prevalence of catastrophic costs due to TB (diagnosis and treatment) among the ACF group was lower than in the PCF group, Morishita et al. did not find any significant differences (36.1% versus 45.0%, *p* = 0.244). This could be because of the low sample size (around 100 in each arm) and lack of a confounder-adjusted analysis. However, they did not provide catastrophic costs due to the TB diagnosis [23].

Similarly, we identified many studies in the recent period that determined catastrophic costs due to TB care (diagnosis and treatment), but did not provide data specifically for diagnosis [11,33–36]. A systematic review from Africa (studies from 1990 to 2010) revealed that the pre-diagnostic costs for TB varied between 10.4 and 35% of pre-TB AHI [12]. Before TB treatment, indirect costs predominate, and medical costs contribute to most of the direct costs [8]. As we collected information on income loss for the time spent on consultation and not for the work absenteeism due to illness, the contribution of indirect costs to total costs was low (<10%). For this reason, our estimate of catastrophic costs due to TB diagnosis is a conservative one.

The total costs incurred were similar across income quintiles. As a result of this, total costs as a proportion of pre-TB AHI and catastrophic costs were concentrated in the poorest two income quintiles. These findings are consistent with findings globally [8,37].

Although ACF had an effect on both total costs and catastrophic costs, it did not reduce the inequity in their distribution. ACF was associated with more inequity in the distribution of total costs as a proportion of pre-TB AHI (when compared with PCF). This could be explained by the fact that the households of patients detected through ACF had a significantly lower MIPC than through PCF (Table 2).

### Implications for policy and practice

Project *Axshya* is planning to continue *Axshya SAMVAD* in the next phase from 2018 to 2020. India’s national strategic plan to eliminate TB (2017–2025) recommends ACF among clinically, socially and occupationally vulnerable populations over and above the existing PCF strategies under RNTCP [38]. This study provides evidence in support of this.

However, there is a need to address the issue of inequity, irrespective of whether TB patients are identified by ACF or PCF. Globally, it is estimated that even with aggressive expansion of TB services (includes multi-drug resistant TB services and ACF), catastrophic costs would decrease only by 5–20% by 2035 (base year: 2015) [39]. Hence, countries need to move towards universal health coverage and social protection. Universal health coverage is expected to reduce the direct medical costs, and social protection is expected to protect against direct non-medical and indirect costs [3–7].

India has proposed a national health protection scheme that would protect 100 million poor households against catastrophic costs with an annual cover of ≈US$7800. However, this is only for hospitalisations and might have a limited effect on reducing costs due to TB diagnosis [40]. India might take lessons from China where, despite social insurance schemes, the catastrophic costs due to TB have decreased marginally with no effect on inequity. One of the reasons was limited outpatient costs coverage [35,37,41].

Under social protection, Rudgard et al. [42] suggested that a TB-specific approach (cash transfers for households with a confirmed case of TB) is more effective and affordable than a TB-sensitive (cash transfers for households with a high TB risk to strengthen their economic resilience) approach to reduce TB-specific catastrophic costs. The Government of India has announced the implementation of direct benefit transfer of ≈US$8 per month up to treatment completion for all patients notified with TB (TB-specific approach) [3,38,42,43]. Further research is recommended to assess the benefits of TB-specific cash transfers on catastrophic costs due to TB, including inequity in India.

## Conclusion

This study highlights the importance of ACF among the marginalised and vulnerable population in mitigating catastrophic costs due to TB diagnosis. ACF among marginalised and vulnerable populations reduced total costs and prevalence of catastrophic costs. However, ACF did not address intensity and inequity in catastrophic costs. This signals a need for implementation of universal health coverage and social protection, in addition to ACF, which will benefit the poorest of the poor. India has taken steps in the right direction, and this needs to be closely monitored if India has to meet the End TB target of ‘zero’ catastrophic costs due to TB, 10 years before the global target of 2035 [3,38].

**Box 1.** Operational definitions of various costs and indicators used in this study [2,30].

TB diagnosis	From eligibility for sputum examination to TB diagnosis. All costs incurred were collected for this period
Direct costs	The sum of the direct medical and direct non-medical costs
Direct medical costs	Costs of consultation fee, medical examinations/investigations and medicines (includes allopathic, traditional system of medicine, paramedical staff, quacks)
Direct non-medical costs	TB diagnosis-related transport. We did not include food and stay costs assuming most of costs due to TB diagnosis would be on outpatient basis
Indirect costs	Patient’s income loss for the time spent on consultation. We calculated the time spent in hours from leaving the home, receiving consultation and returning to home/work. Based on monthly income per capita, assuming 22 working days per month and eight working hours per day, we calculated the value in terms of money for each hour spent for consultation. We did not include income loss from absenteeism from work due to illness
Total costs	Direct plus indirect costs
Total costs as a proportion of annual household income	This indicates the proportion of pre-TB annual household income that went into costs due to TB diagnosis. The advantage of this indicator is that it looks at total costs in relation to the pre-TB annual household income. Household A could have higher total costs than Household B. But if the income of Household A is higher than that of Household B, it could be possible that this indicator could be higher in Household B
Prevalence of catastrophic costs	Number of households whose total costs as a proportion of pre-TB annual household income exceeds 20% divided by the total number of households
Intensity of catastrophic costs	Median positive overshoot from the threshold (20%). Positive overshoot was calculated among those with catastrophic costs by subtracting 20% from the total costs expressed as a proportion of pre-TB annual household income. This indicator captures the extent to which the costs were catastrophic and not just whether they were catastrophic or not

## Paper context

Patients incur significant direct medical, direct non-medical and indirect costs due to TB care. Community-based active case finding for Tuberculosis in marginalised populations reduced total costs and catastrophic costs (more than 20% of AHI) due to TB diagnosis. It did not address the intensity of catastrophic costs and inequity. This calls for implementation of universal health coverage and social protection, in addition to active case finding, which will benefit the poorest of the poor.
