# Supplementary material for: Active case finding among marginalised and vulnerable populations reduces catastrophic costs due to tuberculosis diagnosis
Source: Glob Health Action. 2018 Sep 3;11(1):1494897. doi: 10.1080/16549716.2018.1494897 (PMC6129780; doi:10.1080/16549716.2018.1494897)
Supplement: Supplemental Material [file ZGHA_A_1494897_SM8882.zip › S1 Annex.pdf]

Axshya SAMVAD (Sensitization and Awareness in Marginalised and Vulnerable Areas of the District)

Introduction:

It is believed that TB symptomatics (cough ≥2 weeks) will visit doctor for consultation and treatment. Due to lack of awareness about TB in communities, many at times the diagnosis of TB is missed. As a result, many of TB patients remain undiagnosed and untreated continue to infect community. In-order to overcome late diagnosis and reaching the missing 3 million cases globally, "Active Case Finding" strategy is introduced. "Active Case Finding" strategy involves, one time cross sectional house-to-house visit among high risk community groups to identify TB symptomatics and link them to services. In addition to ACF, the project will also create awareness on the TB. Active case finding for the project is named as **Axshya SAMVAD** (***Axshya Sensitization and Awareness in Marginalised and Vulnerable Areas of the District***)

What is the objective?

Primary objective: To visit the houses of marginalised and vulnerable communities, inform them about TB, its symptoms, diagnosis, treatment and RNTCP services.

Secondary objective: To link the identified TB symptomatic and diagnosed TB patients to RNTCP services through

- Referral and/or
- Collection and transportation of sputum samples to the DMC for diagnosis and/or
- treatment

Where?

This activity will be done in areas identified as marginalised and/or vulnerable in the project. The marginalized and/or vulnerable populations are;

1. Slums,
2. Tribal areas,
3. SC communities,
4. In pockets where Occupational Lung Diseases (OLD) are high
5. In pockets where high risk of acquiring TB like; stone crushing/mining/weaving industry/unorganized labour (construction workers etc)/homeless people
6. In pockets reported to have high HIV/ AIDS burden
7. In areas or communities where incidence of TB is high
8. Among household contacts of sputum smear positive TB patients

What is a household?

A household includes all the persons who occupy a housing unit as their usual place of residence**.** The members of a household eat from the same kitchen**. Eg:** Institutions such as old age homes may not fit into the definition of household.

Who will do this activity?

This activity will be done through Axshya Mithras linked with NGOs or independent Axshya Mitras.

How many households will be reached?

One Axshya Mitra will cover maximum of 25 households per day.

- This activity will be performed in all 285 districts and 40 urban sites.
- Target – 285 districts: 1000 per district per month
- Target – 40 urban sites
    - Target for AS will vary from city to city
    - Targets will be communicated by Project Managers of SR based on slum household population.

When to conduct the activity? Ideally the activity needs to be conducted at the time maximum number of household members are available at home.

What preparation is required before Axshya Samvad?

Planning:

- On a monthly basis, the areas where this activity will be conducted needs to be identified. This should also contain information on the date, the Axshya Mitras involved in the activity (Census data 2011 may be used).
- To attain the objective of AS in a comprehensive manner, Axshya SAMVAD should be conducted in first 15 days of the month and follow up of the TB symptomatics as Referral or SCT in next 10 days and use last 5 days for completion of the reports. This will facilitate the AM to plan and implement AS smoothly in the following month.
- Linkages with Community meetings or other project Axshya activities

Preparation

- 2 days before the intensified outreach activity, the following logistics should be ready
    - The required number of recording formats **(AS 1 and AS 2).**
    - Adequate number of IEC material, referral forms and sputum cups
    - The local community leaders are informed of this activity.
    - Obtain detailed information on where this activity will be done, the number of households to be covered etc.,

Implementation of Axshya SAMVAD:

- Visit the households serially up to a maximum of 25 households per Axshya Mitra per day. If more than 25 houses are covered by same AM on the same day, it will not be considered for payment.
- Obtain verbal consent from the head of the household or the senior member of the household for continuing with the conversation.

- After obtaining verbal consent, please request that all members of the household present at that time must join and listen to the conversation

- ***The following key information/ messages must be provided***

  (A minimum of 15 minutes has to be spent at each of the household.)

  - TB is an infectious airborne disease caused by germs
  - TB can happen to anyone
  - The key symptom is cough of 2 weeks or more. The other symptoms include fever, chest pain, loss of appetite, loss of weight, night sweats, blood in sputum etc.,
  - People with cough of 2 weeks or more must get their sputum examination done in a nearest government health facility (the name and address of the nearest Designated Microscopy Centre has to be provided)
  - It is completely curable with 6-8 months of regular TB treatment.
  - Sputum examination and full course of TB treatment is provided free of cost by the Government Health facilities under DOTS
  - Relevant IEC material with key messages on TB to be used at Every House
    - for sensitization (IPC tool kit, small flip chart etc.)
    - to be given to family members (IPC leaflet) .

- After this information is provided there must be a provision of answering any questions that the household members may have.
- Then specific questions need to be asked whether any member of their household had TB in the past? (note in remarks column)
- Further, it must also be asked whether any member of the household currently has cough of 2 weeks or more or any other related symptom of TB. If yes then such persons need to be referred to the nearest DMC. If required, sputum collection and transportation needs to be organised.
- In case there are any children < 6 years in the household who are suffering from fever **or** cough **or** loss of weight **or** loss of appetite of > 2 weeks duration, they should be referred to the nearest health centre for further investigations for TB.
- It is the responsibility of the NGO representative/ AM to link TB Symptomatics or TB patients to RNTCP diagnostic and treatment services. **This may be achieved through repeat follow-up visits/ and or Telephone calls.**
- **The form AS 1 should be filled immediately after completion of each house**. The format should not be changed and all sections to be filled. Incomplete forms will be rejected. At least one side of each paper of AS 1 should be with top information. (place village, district, block, name of the CV, date, reason for conducting AS in this locality etc.)
- The house covered under Axshya SAMVAD to be marked using permanent marker pens and this is mandatory. Marking could be made on wall, door, window, any wood material visible in front

of the house. Information under marking could be related to Axshya, SR, NGO implementing this activity with compulsory Number of HH and Date.

- Example marking for first house as : AX/SR/001

  DD/MM/YY

- If the household is locked or if there are no adults (at the time of visit) to the household then the household should not counted.
- If any person is found to be symptomatic then fill in the details on **AS 2.**
- Please note that the information collected at the household level on TB patients or TB symptomatics must be kept confidential and not discussed with any other member of the community.

After the day of the Axshya SAMVAD

- Within the next 2 days check if the persons referred have reached the DMC or not. **Tracking of each TB symptomatic is responsibility of the AM** who had conducted AS. Tracking of these TB symptomatic could be physical or telephonic follow up. If the referred TB symptomatics have not reached the DMC then arrange for sputum collection and transportation.
- Within 10 days of completing the Axshya SAMVAD, all details of **AS 2** (with necessary laboratory information, TB diagnosis and treatment initiation) have to be filled up.
- **AS 1, AS 2** along with copy of referral slips (triplicate) has to be pinned/stapled together and submitted to the NGO/ District Coordinator for financial reimbursement/ Settlement.

Recording and reporting: It is expected that the NGO and/or community AM will conduct this activity and provide the details in the format **AS 1 – Axshya SAMVAD**. If they come across any TB symptomatics then they will have to make a referral of such persons using the referral books/slips that they currently use. The counter foil of the referral made must be retained with the NGO partner/ AM for verification. If sputum is collected and transported, it should be as per guidelines (spot and early morning samples) and the details will have to be recorded in the format that they currently use for this activity. For all TB symptomatics that they refer, they must be able to provide details on how many reached the DMC (with DMC name and lab numbers), how many sputum positive/sputum negative TB cases were diagnosed and how many of those diagnosed were initiated on TB treatment **(AS 2 – Axshya SAMVAD)**. It should be ensured that at least 80% of the TB symptomatics identified through this activity get tested at the DMCs within a week of conducting the activity. **(Please note that our objective is to inform the households about TB and RNTCP services and to link TB symptomatics and TB patients in these populations to RNTCP services).**

Supervision and monitoring: This activity will be supervised and monitored by the DC/IPCC/UC, APM, MC, PM, ACSM consultants and the technical officers from The Union PMU.

- DC/IPCC/UC **to verify Axshya SAMVAD conducted by each NGO/AM every quarter through the following methods:**
  - During conducting AS

- Physically after completion of AS
- through telephone after completion of AS

Based on the verification, DC/IPCC/UC to report under AS 3 for further processing payment.

- The APM, MC, PM, ACSM Consultants and technical officers from The Union PMU whenever they visit the district, will select areas/villages 'randomly' where this activity has been conducted during the last 3 months and visit these areas.
- During the visit, the DC,IPCC,UC,APM,PM,MC,ACSM consultants and technical officers from The Union PMU will cross verify the following
  - Whether the areas chosen for conducting this activity is appropriate or not? (i.e., whether the area belongs to designated marginalised and vulnerable communities?)
  - Whether the activity was conducted in the locality or not?
  - Whether the households were able to recall the key messages that were communicated by the NGO/AM during this activity?
  - Whether the TB symptomatics identified during this activity have undergone sputum examination at RNTCP DMCs and if diagnosed with TB are they being treated by RNTCP medications or not?

They will report their observations and recommendations in their supervisory visit report of SRs and the same communicated to respective DC/IPCC/UC of the district.

The following points should be adhered to while conducting the Axshya SAMVAD: (at the district level per month)

- What proportion of the households visited belonged to vulnerable and marginalised populations (> 90% households visited must be from identified vulnerable and marginalized communities)
- How many TB symptomatics were identified? (Assuming 0.5 – 1 % prevalence of TB symptomatics in the general population, it is expected that at-least 20 to 40 TB symptomatics will be present per 1000 HHs. All identified should be referred/ sputum collected and transported to the nearest DMC).
- How many diagnosed TB patients were initiated on RNTCP? (All TB patients are expected to be initiated on RNTCP treatment)

AS 1: Household information of Axshya SAMVAD

[Please take the printout back to back on an A4 Sheet of paper. Each single sheet (back to back) to have details of 25 household]

Name of the village/ward: **Name of the Block:** **Name of the District/Urban site:**

Date of the activity (dd/mm/yyyy): **Name of the person conducting the activity:**

Type of KAP covered: (circle the appropriate type):

(1) Slum (2) SC (3) Tribal (ST) (4) Prisons (5) Unorganised labour (UL) (6) PLHIV (7) Contacts of TB patients (8) Occupational Lung Disease (OLD) (9) Hard to reach (HtR)

Sl No.	House No.	Household (HH) head's name	Telephone number	Total number of household members		Number of HH members having cough of $\geq$2weeks	Remarks
				Adults	Children $\leq$14 years		
a	b	c	d	e	f	g	h

AS 2: Status of TB symptomatics identified during Axshya SAMVAD
[Please take the printout back to back on an A4 Sheet of paper. Each single sheet (back to back) to have details of 25 patients]

Name of the village/ward: **Name of the Block:** **Name of the District/Urban site:**

Date of the activity (dd/mm/yyyy): **Name of the person conducting the activity:**

Sl No/ HH No. As per AS 1	Name of the TB symptomatic identified	Age (in yrs)	Sex M/F	Telephone number	Type of KAP**	Referred / Sputum C &T (R/S)	Date of Referral / Sputum C &T	Name of DMC	Date of sputum examination at DMC and lab number	Sputum result (Pos/Neg)	Has the patient been diagnosed as smear neg TB (Yes/No)	Date of starting treatment	Remarks*
a	b	c	d	e	f	g	h	i	j	k	l	m	n

(*write reasons in the remarks column if the person has not undergone sputum examination or is not initiated on treatment)
**KAP (1) Slum (2) SC (3) Tribal (ST) (4) Prisons (5) Unorganised labour (UL) (6) PLHIV (7) Contacts of TB patients (8) Occupational Lung Disease (OLD) (9) Hard to reach (HtR)

AS 3: Monthly Summary Report of Axshya SAMVAD

Name of the Month:

Name of the district/urban site:

Name of the DC/IPCC/UC:

Sl.No.	Particulars	Slum	SC	ST	UL	Prison	PLHIV	Contacts	OLD	HtR	Total
a	b	c	d	e	f	g	h	i	j	k	l
1	Total no. of HHs visited										
2	Total no. of HH members										
3	Total Number of IPC leaflets distributed										
4	Total Number of TB Symptomatics identified and referred										
5a	Referral reached DMC										
5b	SCT										
5c	Total tested at DMC (5a+5b)										
6	Number of TB patients diagnosed (all forms*)										
7	Number initiated on treatment										

*all forms will include smear positive, smear negative, extra pulmonary

Supervision/Verification of AS by DC/IPCC/UC

Sl. No.	Name of the Village	No. of HHs supervised while conducting AS		No. of HHs verified physically after completion of AS		No. of HHs verified through telephone		Remarks
		No. of HHs	Sl. No. of HHs of AS1	No. of HHs	Sl. No. of HHs of AS1	No. of HHs	Sl. No. of HHs of AS1	
a	b	c	d	e	f	g	h	i

- Sl. No. of AS 1 verified-mention the sl. No. of the HHs as recorded in the AS1 format
- DC /IPCC/UC need to verify minimum 10 HHs in each category per NGO in a quarter (Take the total of all the 3 monthly AS -3 reports of the respective quarter for this purpose)
- This format should be used by APM/MC/PM
