# Supplementary material for: Active case finding among marginalised and vulnerable populations reduces catastrophic costs due to tuberculosis diagnosis
Source: Glob Health Action. 2018 Sep 3;11(1):1494897. doi: 10.1080/16549716.2018.1494897 (PMC6129780; doi:10.1080/16549716.2018.1494897)
Supplement: Supplemental Material [file ZGHA_A_1494897_SM8882.zip › S2 Annex.pdf]

AXSHYA SAMVAD STUDY _ PART I of THE QUESTIONNAIRE _ RECORD REVIEW   **Date of enrolment (dd/mm/yyyy)-**

Annexure 1: Data Collection Instrument / Questionnaire

(PLEASE FILL USING CAPITALS LETTERS; DO NOT LEAVE ANY FIELD BLANK, MENTION MISSING)

Part I: Patient level Data to be collected from registers/project forms/electronic project data (District Coordinator, Project Axshya)

Note: - Before including the patient into the study, confirm that the patient is New Sputum Positive Pulmonary TB

Sno	Variable	Source of data / possible values	Value
1	Unique id	(TUCODE_tbregyr_tbregno)	
2	Exposure status of patient	Exposed-AS; Unexposed-NonAS Axshya; Unexposed-NonAxshya	
3	Date of data collection	Dd/mm/yyyy	
4	TU name and TUCODE		
5	Year of registration for TB treatment (tbregyr)	TB treatment register	
6	TB registration number (tbrego)	TB treatment register	
7	Name of patient<br>**Mobile number** of the patient	TB treatment register	
8	Name of district		
9	Name of Block / City	From address of patient TB treatment register	
10	Name of village/ Mohalla	From address of patient TB treatment register	
11	Whether urban or rural	DC to interpret from address of patient	
12	Distance (km) of Village/Mohalla from nearest PHC or Urban Dispensary	To interpret from address of patient, if required use google map	
13	Distance (km) of Village / Mohalla from nearest DMC	Same as above if DMC is located in the nearest PHC/Disp.	
14	Age in completed years	TB treatment register	
15	Sex	TB treatment register (male/female/transgender)	
16	DMC where sputum examination/diagnosis was done	TB treatment register	
17	Date of sputum examination (date of diagnosis)	TB treatment register (dd/mm/yyyy)	
18	Sputum test result at diagnosis	Scanty+ / 1+ / 2+ / 3+	
19	Weight at baseline	TB treatment card	
20	HIV status	TB treatment register (positive/negative/unknown)	
21	Diabetes Mellitus	(positive/negative/unknown)	
22	Date of treatment initiation	TB treatment register (dd/mm/yyyy)	
	**If the patient exposure status is exposed-AS, then note the following details; else mention Not Applicable (NA)**		
23	Date of Axshya SAMVAD activity	AS1/AS2 form (dd/mm/yyy)	
24	Patient AS status	AS1/AS2 form Referred / SCT	
25	Date of referral / SCT (whichever was the final mode of sputum testing)	AS1/AS2 form (dd/mm/yyy)	
26	Serial number in AS1form	AS1 form	
27	House Number in AS1 in form	AS1 form	
