# Supplementary material for: Active case finding among marginalised and vulnerable populations reduces catastrophic costs due to tuberculosis diagnosis
Source: Glob Health Action. 2018 Sep 3;11(1):1494897. doi: 10.1080/16549716.2018.1494897 (PMC6129780; doi:10.1080/16549716.2018.1494897)
Supplement: Supplemental Material [file ZGHA_A_1494897_SM8882.zip › S3 Annex.pdf]

AXSHYA SAMVAD STUDY _ PART II of THE QUESTIONNAIRE _ PATIENT INTERVIEW

Date of enrolment (dd/mm/yyyy)-

Annexure 1: Data Collection Instrument / Questionnaire
(PLEASE FILL USING CAPITALS LETTERS; DO NOT LEAVE ANY FIELD BLANK; MENTION MISSING)

Note: - Before starting the interview, confirm that the patient is New Sputum Positive TB (to confirm correct categorization as per records) by taking a history of previous TB treatment. If history suggests otherwise, i.e., not new sputum positive (past history of ATT for more than a month either from Govt or Pvt present), further interview should not be done; make a note of the same in the form and send it across along with other forms.
Note:- Before starting the interview go through Part I of the same patient and fill enrolment date, unique id and date of diagnosis from Part I.

Part II: Patient level data to be elicited form the patient (rater administered interview – ACSM consultant)

EDUCATION, OCCUPATION, INCOME, DELAYS, OTHER BASELINE VARIABLES

Sno	Variable	Source of data / possible values	Value
1	Unique id	(TUCODE_tbregyr_tbregno)	
	Date of TB diagnosis / sputum examination	Dd/mm/yyyy. To be entered in the form before itself by contacting the DC from Part I. We will depend on the lab / treatment register rather than patient for this data. This data from part II is not to be entered during data entry. This date will help you to derive the smoking / alcoholic status of the patient.	
2	Date of interview	dd/mm/yyyy	
3	Highest education of the patient	Codes below	
4	Highest education of Head of Household	Codes below	
5	Occupation of Patient	Codes below	
6	Occupation of Head of Household	Codes below	
7	Monthly household income per capita (INR)	Total income of all household members per month **divided by total number of household members**. (mention unknown if the patient does not know)	
8	Any patient of TB in household ever	Yes / No	
9	Any TB death in the household	Yes / No	
10	Previous history of TB in the patient	Yes/ No	
11	Date when eligible for sputum AFB (approximate, elicit from history)	Dd/mm/yyyy<br>This is the approximate date from when the patient was eligible for sputum AFB. 15th day of continuous cough or 15th day of fever or 1st episode of haemoptysis or onset of weight loss (whichever is earlier would be the 'date when eligible for sputum AFB')	
12	History of fever between date of eligibility and	Yes/no/Cannot say	

	date of sputum examination		
13	History of hemoptysis between date of eligibility and date of sputum examination	Yes/No/cannot say	
14	History of significant weight loss (as perceived by the patient) between date of eligibility and date of sputum examination	Yes/No/cannot say	
15	Whose advice led to sputum examination?	1 Community Member 2 MPW/ANM 3 RHCP<br>4 Govt doctor 5 Pvt Doctor 6 family member 7 Axshya Mitra<br>8 Someone else (specify)	
16	Date when first visited a health care provider after being eligible for sputum AFB	Dd/mm/yyyy	
17	Type of health care provider first visited after being eligible for sputum AFB	1 qualified 2 quack/unqualified	
18	If qualified, the type of health care provider-1	Govt. doctor (qualified), Pvt doctor (qualified), RHCP (quack), ANM/ASHA/MPHW/AWW, Others (specify)	
19	Number of health care providers visited before TB diagnosis (Do cross check this number with the table below)*	Include first health care provider visited, include the final visit to DMC when sputum examination was done and TB diagnosed. *Do note that for 'AS – exposed' patients who were referred through SCT, the patient didn't have to visit the DMC.*	
20	Alcoholic at diagnosis **	Yes/No	
21	Smoker at diagnosis **	Yes/No	

Education Codes: *0 Cannot read or write / 1 Can read write, not completed primary school / 2 Primary School (class 5) completed*
3 Middle School(class 8) completed / 4 Secondary School (class 10) / 5 Senior Sec (class 12), Graduate, Degree, Diploma, ITI, Professional
Occupation Codes: *1 Studying / 2 Household work only (females) / 3 Unemployed / 4 Daily wage labourer / 5 Agriculture / animal rearing*
6 Service / 7 Shopkeeper / 8 Business / 9 Professional. If there is any change of occupation, please consider occupation as on the date of diagnosis

**Number of HCPs (not consultations). Ignore additional visits or consultation with the sae HCP.*
***Yes if consumed alcohol / smoked tobacco within 30 days from the date of diagnosis / sputum examination*

Health Care Provider (HCP) include qualified allopathic (Govt or private), qualified medical AYUSH (Govt or private), qualified paramedical (ANM, ASHA, AWW, LT, pharmacist, MPW) and unqualified RHCP. If a Govt doctor does private practice and the patient meets him/her in his/her private clinic then it will be considered as 'private' HCP. If a Govt doctor does private practice and the patient meets him/her in his/her Govt clinic then it will be considered as 'Govt' HCP.

Date of enrolment (dd/mm/yyyy)-

TREATMENT SEEKING BEHAVIOUR AND COSTS

(include the final visit to DMC during which sputum was tested and diagnosis made) **Unique id:**

Try and remember from which all sources you have taken treatment for TB symptoms. Then, one by one starting from the first treatment provider, I would like to know who all were consulted, when did you consult, how many consultations did you have, and how much did you have to spend for consultations, medicines, investigations, and travel. Finally, I would like to know how many days were you away from work.

Do note that for 'AS – exposed' patients who were referred through SCT, the patient didn't have to visit the DMC

	Who was consulted? (Use codes below)	Date of consultation	After how many days/ wks / mths of 'the date when eligible for sputum AFB' was this HCP consulted?	How much money did you have to spend in total				In total, how much time (hours) did it take for you from the time you left house and you came back to home/work from this Rx provider
				Each consultation (INR)	Medicines (INR)	Investigations (INR)	travelling (coming and going back) (INR)*	
**Sno**	**19**	**20**	**21**	**22**	**23**	**24**	**25**	**26**
i			D / w/ m / y					
ii			D / w/ m / y					
iii			D / w/ m / y					
iv			D / w/ m / y					
v			D / w/ m / y					
vi			D / w/ m / y					
vii			D / w/ m / y					
viii			D / w/ m / y					
ix			D / w/ m / y					
x			D / w/ m / y					

CODES 1 Government Doctor (qualified) 2 Private Doctor (qualified) 3 RHCP (quack) 4 ANM/MPW/ASHA 5 Other (Specify) (mention repeat consultations to the same health care provider as separate entries/consultations)

**to maintain uniformity: 5 Rs per km for personal two wheeler, 10 Rs per km for personal three/four wheeler *information collected regarding qualification of HCP (qualified or unqualified) will be confirmed by contacting the project staff working in the district (IPCC or DC)*
