# Supplementary material for: Active case finding among marginalised and vulnerable populations reduces catastrophic costs due to tuberculosis diagnosis
Source: Glob Health Action. 2018 Sep 3;11(1):1494897. doi: 10.1080/16549716.2018.1494897 (PMC6129780; doi:10.1080/16549716.2018.1494897)
Supplement: Supplemental Material [file ZGHA_A_1494897_SM8882.zip › S4 Annex_Investigator names and affiliations.docx]

**This is a multi-author study (n=50).**

**Before submitting the manuscript, I raised this query with “Academic Journals ZGHA Peer Review** [**ZGHA-peerreview@tandf.co.uk**](mailto:ZGHA-peerreview@tandf.co.uk)**”. I asked whether I needed to enter details of all the authors in the manuscript submission website? (mail from** [**hemantjipmer@gmail.com**](mailto:hemantjipmer@gmail.com) **dated 9 April 2018).**

**I received a response from Nicola Parsons (mail** [**ZGHA-peerreview@tandf.co.uk**](mailto:ZGHA-peerreview@tandf.co.uk) **dated 13 April 2018) that I submit just the details of the first / corresponding author in the manuscript submission website and submit a supplementary file with the manuscript listing all of the other authors.**

**Investigator names and affiliations**

*Axshya* *SAMVAD* study group: Hemant Deepak Shewade^1*^, Vivek Gupta^2^, Srinath Satyanarayana^3^, Atul Kharate^4^, KN Sahai^5^, Lakshmi Murali^6^, Sanjeev Kamble^7^, Madhav Deshpande^8^, Naresh Kumar ^9^, Sunil Kumar^10^, Prabhat Pandey^1^, UN Bajpai^11^, Jaya Prasad Tripathy^1^, Soundappan Kathirvel ^1,12^, Sripriya Pandurangan^1^, Subrat Mohanty^1^, Vaibhav Haribhau Ghule ^1^, Karuna D Sagili^1^, Banuru Muralidhara Prasad^1^, Sudhi Nath^1^, Priyanka Singh^13^, Kamlesh Singh^14^, Ramesh Singh^11^, Gurukartick Jayaraman^15^, P Rajeswaran^15^, Binod Kumar Srivastava^16^, Moumita Biswas^1^, Gayadhar Mallick^1^, Om Prakash Bera^1^, A James Jeyakumar Jaisingh^15^, Ali Jafar Naqvi^13^, Prafulla Verma^13^, Mohammed Salauddin Ansari^16^, Prafulla C Mishra^17^, G Sumesh^15^, Sanjeeb Barik^18^, Vijesh Mathew^14^, Manas Ranjan Singh Lohar^18^, Chandrashekhar S Gaurkhede^14^, Ganesh Parate^13^, Sharifa Yasin Bale^14^, Ishwar Koli^14^, Ashwin Kumar Bharadwaj^14^, G Venkatraman^15^, K Sathiyanarayanan^15^, Jinesh Lal^14^, Ashwini Kumar Sharma^16^, Raghuram Rao^19#^, Ajay MV Kumar^1,3#^, Sarabjit Singh Chadha^1#^

^#^RR, AMVK and SSC contributed equally as senior authors

1. International Union Against Tuberculosis and Lung Disease (The Union), South-East Asia Office, New Delhi, India - 110016
2. All India Institute of Medical Sciences (AIIMS), New Delhi, India - 110029
3. International Union Against Tuberculosis and Lung Disease (The Union), Paris, France – 75006
4. State TB Cell, Department of Health & Family Welfare, Government of Madhya Pradesh, Bhopal, India - 462004
5. State TB Cell, Department of Health & Family Welfare, Government of Bihar, Patna, India - 800015
6. State TB Cell, Department of Health & Family Welfare, Government of Tamil Nadu, Chennai, India - 600006
7. State TB Cell, Health Department, Government of Maharashtra, Pune, India - 411006
8. State TB Cell, Department of Health & Family Welfare, Government of Chattisgarh, Raipur, India - 492002
9. State TB Cell, Department of Health & Family Welfare, Government of Punjab, Chandigarh, India – 160022
10. State TB Cell, Department of Health & Family Welfare, Government of Kerala, Thiruvananthapuram, India - 695 035
11. Voluntary Health Association of India (VHAI), New Delhi, India - 110016
12. Post Graduate Institute of Medical Education and Research (PGIMER), Chandigarh, India – 160012
13. MAMTA Health Institute for Mother and Child, New Delhi, India - 110048
14. Catholic Health Association of India (CHAI), Telangana, India - 500009
15. Resource Group for Education & Advocacy for Community Health (REACH), Chennai, India – 600014
16. Population Services International (PSI), New Delhi, India - 110019
17. Catholic Bishops’ Conference of India-Coalition for AIDS and Related Diseases(CBCI-CARD), New Delhi, India - 110001
18. Emmanuel Hospital Association (EHA), New Delhi, India – 110019
19. Central TB Division, Revised National Tuberculosis Control Programme, Ministry of Health and Family Welfare, Government of India, New Delhi, India – 110011

**Email id of all authors**

Hemant Deepak Shewade - hemantjipmer@gmail.com

Vivek Gupta - drvivek.gupta@gmail.com

Srinath Satyanarayana - drsrinaths@gmail.com

Atul Kharate - drkatul@yahoo.co.in

K N Sahai - dr.knsahai@gmail.com

Lakshmi Murali - lak_murali668@yahoo.co.in

Sanjeev Kamble - drsanjivkamble@rediffmail.com

Madhav Deshpande - drmadhavdeshpande@gmail.com

Naresh Kumar - sharma.drnaresh@yahoo.in

Sunil Kumar - sunilkumarm2000@gmail.com

Prabhat Pandey - dr.prabhatpandey@gmail.com

U N Bajpai - unbajpai.2010@gmail.com

Jaya Prasad Tripathy - ijay.doc@gmail.com

Soundappan Kathirvel - selvkathir@gmail.com

Sripriya Pandurangan - sripriya14@gmail.com

Subrat Mohanty - subratmohanty1965@gmail.com

Vaibhav Haribhau Ghule - drvaibhavghule@gmail.com

Karuna D Sagili - drkarunas@gmail.com

Banuru Muralidhara Prasad - drprasadbm@gmail.com

Sudhi Nath - drsudhinath@gmail.com

Priyanka Singh - singh.pinkal@gmail.com

Kamlesh Singh - puymsgwl@gmail.com

Ramesh Singh - rameshcamp@rediffmail.com

Gurukartick Jayaraman - gurukart@gmail.com>

P Rajeswaran - vkprajes@gmail.com

Binod Kumar Srivastava - binod_cpr8@rediffmail.com

Moumita Biswas - moumita2209@gmail.com

Gayadhar Mallick - gayadharmallick@yahoo.com

Om Prakash Bera - dromprakashberapgi@gmail.com

A James Jeyakumar Jaisingh - james.jaising@gmail.com

Ali Jafar Naqvi - alijafar.naqvi@gmail.com

Prafulla Verma - mailmeprafulla@rediffmail.com

Mohammed Salauddin Ansari - mdsalahukrg@gmail.com

Prafulla C Mishra - pcmishra2002@gmail.com

G Sumesh - reachsumeshg@gmail.com

Sanjeeb Barik - bsanjeeb2007_msw@yahoo.co.in

Vijesh Mathew - vijeshmt@gmail.com

Manas Ranjan Singh Lohar - lohar.manas@gmail.com

Chandrashekhar S Gaurkhede - shekhar7551@gmail.com

Ganesh Parate - ganeshparate@yahoo.com

Sharifa Yasin Bale - sharifabale@gmail.com

Ishwar Koli - ishwarkoli@gmail.com

Ashwin Kumar Bharadwaj - ashi7566@gmail.com

G Venkatraman - venkatreach2014@gmail.com

K Sathiyanarayanan - biosathya@gmail.com

Jinesh Lal - rvjineshlal@gmail.com

Ashwini Kumar Sharma - ashwinicalling@gmail.com

Raghuram Rao - drraghuramrao@gmail.com

Ajay M V Kumar - sathyasaakshi@gmail.com

Sarabjit S Chadha - drsschadha@gmail.com
