# Supplementary material for: Active case finding among marginalised and vulnerable populations reduces catastrophic costs due to tuberculosis diagnosis
Source: Glob Health Action. 2018 Sep 3;11(1):1494897. doi: 10.1080/16549716.2018.1494897 (PMC6129780; doi:10.1080/16549716.2018.1494897)
Supplement: Supplemental Material [file ZGHA_A_1494897_SM8882.zip › Supplementary tables_catas.docx]

**S1 Table. Unadjusted association (p value^) of potential confounders with *Axshya* *SAMVAD* exposure (exposure of interest) and catastrophic costs due to TB diagnosis (outcome), *Axshya* *SAMVAD* study, India, 2016-17(n=465)**

| **Potential confounder** | ***Axshya* *SAMVAD* exposure** | **Catastrophic costs due to TB diagnosis** |
| --- | --- | --- |
| Age in years | 0.003 | 0.394 |
| Gender | 0.721 | 0.886 |
| Residence (urban/rural) | <0.001 | 0.611 |
| Education | <0.001 | 0.075 |
| Occupation | 0.283 | 0.001 |
| Monthly income per capita | 0.001 | <0.001 |
| TB in household (ever) | 0.321 | 0.175 |
| TB death in household (ever) | 0.704 | 0.519 |
| History of fever | 0.231 | 0.015 |
| History of hemoptysis | 0.937 | 0.011 |
| History of significant weight loss | 0.032 | 0.005 |
| Alcohol intake | 0.419 | 0.283 |
| Smoking | 0.122 | 0.710 |
| Weight categories | 0.540 | 0.721 |
| Diabetes | 0.784 | 0.361 |
| Severe sputum grading (3+ or not) | 0.068 | 0.318 |
| Distance from nearest DMC in km | 0.090 | 0.037 |

TB – tuberculosis; *SAMVAD* – sensitization and advocacy in marginalised and vulnerable areas of the district; *Axshya SAMVAD* – an active case finding strategy under project *Axshya* implemented by The Union, South East Asia office, New Delhi, India, across 285 districts of India

^For association. mann whitney U test or unpaired t test or Chi square test was used;

**S2 Table.** **Comparison of baseline characteristics among study participant whose structured one-to-one interview (part II of questionnaire) was conducted and not conducted, *Axshya* *SAMVAD* study, India, April 2016 – Mar 2017 (N=573)**

| **Variable** | **Interview conducted (n=465)** | **Interview not conducted (n=108)** | **P value** |
| --- | --- | --- | --- |
| Exposed to *Axshya* *SAMVAD* [n (%)] | 234 (50) | 42 (39) | 0.03* |
| Rural residence [n (%)] | 402 (87) | 82 (77) | <0.01* |
| Distance of residence from DMC [Median (IQR)] | 10 (5,15) | 10 (4,15) | 0.92^ |
| Age [Mean (SD)] | 42 (17) | 40 (18) | 0.31** |
| Male gender [n (%)] | 307 (66) | 70 (65) | 0.88* |
| Sputum result 3+ at diagnosis | 83 (18) | 9 (9) | 0.02* |
| Weight in kg at diagnosis Mean (SD)] | 41 (7) | 40 (8) | 0.30** |

Column percentage

*SAMVAD* – sensitization and advocacy in marginalised and vulnerable areas of the district; *Axshya SAMVAD* – an active case finding strategy under project *Axshya* implemented by The Union, South East Asia office, New Delhi, India, across 285 districts of India

Only one study participant was HIV positive; DM status missing in programme records for >60% study participant records

*Chi square test; ^Krushkal Wallis test, **Unpaired t test

**S3 Table. Median (IQR) time taken (in days) for completion of data collection for part I (record review) and part II (patient interview at residence) of the questionnaire after study participant enrolment in *Axshya* *SAMVAD* study across 18 randomly sampled districts in India, April 2016-Mar 2017**

|  | Total (n=573) | *Axshya* *SAMVAD* (n=276) | Non-*Axshya* *SAMVAD* (n=297) | p value |
| --- | --- | --- | --- | --- |
| Part I | 0 (0,7) | 0 (0,8) | 0 (0,7) | 0.70 |
| Part II | 41 (24,62) | 40 (27, 62) | 41 (24, 62) | 0.92 |

*SAMVAD* – sensitization and advocacy in marginalised and vulnerable areas of the district; *Axshya SAMVAD* – an active case finding strategy under project *Axshya* implemented by The Union, South East Asia office, New Delhi, India, across 285 districts of India

Part II was not filled for 108 patients (42 – *Axshya SAMVAD*; 66 – Non-*Axshya SAMVAD*)

Of 465 patients for whom part II was filled, data collection was done within the target 60 days of enrolment in 332 (71.4%) instances

Part I was filled for all: it was done within the target 30 days in 519 (90.6%) instances

**S4 Table. Comparison of median costs (USD) due to TB diagnosis with findings from the study by Morishita F et al (Cambodia, 2012-13).**

|  | **This study, India, 2016-17*** | | **Morishita F et al, Cambodia, 2012-13^** | |
| --- | --- | --- | --- | --- |
| **Costs** | **ACF** | **PCF** | **ACF** | **PCF** |
| **Total costs** | 4.7 | 20.4 | 5.1 | 22.4 |
| **Direct costs** | 4.2 | 19.1 | 2.5 | 14.8 |
| **Indirect costs**  **(Wages / income lost)** | 0.1 | 0.6 | 0.0 | 1.4 |

TB – tuberculosis; ACF – active case finding; PCF – passive case finding; USD – United States dollar

*ACF among marginalised and vulnerable populations

^ACF among household and neighbourhood contacts
